# Reduced β-cell function in early preclinical type 1 diabetes

**DOI:** 10.1530/EJE-15-0674

**Published:** 2016-03

**Authors:** Maarit K Koskinen, Olli Helminen, Jaakko Matomäki, Susanna Aspholm, Juha Mykkänen, Marjaana Mäkinen, Ville Simell, Mari Vähä-Mäkilä, Tuula Simell, Jorma Ilonen, Mikael Knip, Riitta Veijola, Jorma Toppari, Olli Simell

**Affiliations:** 1 Department of Paediatrics, University of Turku and Turku University Hospital, Turku, Finland; 2 MediCity Laboratories, Department of Clinical Medicine, University of Turku, Lemminkäisenkatu 320520, Turku, Finland; 3 PEDEGO Research Unit, Department of Paediatrics, Medical Research Centre Oulu, University of Oulu, Oulu, Finland; 4 Department of Children and Adolescents, Oulu University Hospital, Oulu, Finland; 5 Clinical Research Centre, Turku University Hospital, Turku, Finland; 6 Department of Paediatrics, Tampere University Hospital, Tampere, Finland; 7 Novo Nordisk Farma Oy, CMR Department, Espoo, Finland; 8 Diabetes Outpatient Clinic, Tampere, Finland; 9 Department of Paediatrics, Turku University Hospital, Turku, Finland; 10 Research Centre of Applied and Preventive Cardiovascular Medicine, University of Turku, Turku, Finland; 11 Immunogenetics Laboratory, University of Turku, Turku, Finland; 12 Department of Clinical Microbiology, University of Eastern Finland, Kuopio, Finland; 13 Children's Hospital, University of Helsinki and Helsinki University Hospital, Helsinki, Finland; 14 Research Programs Unit, Diabetes and Obesity, University of Helsinki, Helsinki, Finland; 15 Folkhälsan Research Centre, University of Helsinki, Helsinki, Finland; 16 Department of Physiology, Institute of Biomedicine, University of Turku, Turku, Finland

## Abstract

**Objective:**

We aimed to characterize insulin responses to i.v. glucose during the preclinical period of type 1 diabetes starting from the emergence of islet autoimmunity.

**Design and methods:**

A large population-based cohort of children with HLA-conferred susceptibility to type 1 diabetes was observed from birth. During regular follow-up visits islet autoantibodies were analysed. We compared markers of glucose metabolism in sequential intravenous glucose tolerance tests between 210 children who were positive for multiple (≥2) islet autoantibodies and progressed to type 1 diabetes (progressors) and 192 children testing positive for classical islet-cell antibodies only and remained healthy (non-progressors).

**Results:**

In the progressors, the first phase insulin response (FPIR) was decreased as early as 4–6 years before the diagnosis when compared to the non-progressors (*P*=0.001). The difference in FPIR between the progressors and non-progressors was significant (*P*<0.001) in all age groups, increasing with age (at 2 years: difference 50% (95% CI 28–75%) and at 10 years: difference 172% (95% CI 128–224%)). The area under the 10-min insulin curve showed a similar difference between the groups (*P*<0.001; at 2 years: difference 36% (95% CI 17–58%) and at 10 years: difference 186% (95% CI 143–237%)). Insulin sensitivity did not differ between the groups.

**Conclusions:**

FPIR is decreased several years before the diagnosis of type 1 diabetes, implying an intrinsic defect in β-cell mass and/or function.

## Introduction

Type 1 diabetes is an autoimmune disease leading to the loss of insulin secretion from the pancreatic β-cells, which ultimately results in high circulating plasma glucose levels and clinical symptoms (1). Islet autoantibodies are predictive of the disease, and in children they typically appear within the first years of life (2, 3, 4). Currently, type 1 diabetes is diagnosed at a younger age than previously (5, 6).

One of the most commonly used methods to assess the capacity of the β-cells to secrete insulin is the intravenous glucose tolerance test (IVGTT), where the first 10 min represent the initial burst, the acute insulin response to a rapid glucose stimulus (7). It occurs via the release of insulin from the membrane-docked secretory granules within the β-cells (8). First phase insulin response (FPIR), calculated as the sum of serum insulin concentrations at 1 and 3 min in the IVGTT, has been shown to decline before the diagnosis (9, 10, 11) and a reduced FPIR clearly predicts clinical type 1 diabetes (12, 13, 14, 15). Overall, the loss of FPIR has been considered to be a rather late sign of the disease process and a marker of β-cell pathology. However, when FPIR was analysed shortly after seroconversion in a cross-sectional setting in the Type 1 Diabetes Prediction and Prevention (DIPP) Study, 55% (18 of 33) of children with biochemically defined autoantibodies had reduced FPIR values whereas 21% (four of 19) of those with only islet autoantibodies had decreased FPIR (16). In the Diabetes Prevention Trial-type 1 where the longitudinal pattern of decline in FPIR was analysed, FPIR appeared to decline already between 4.4 and 1.5 years before diagnosis (9). Furthermore, the baseline FPIR was lower in those who progressed to type 1 diabetes than that in the non-progressors, also suggesting early dysfunction of β-cells (9).

The aim of the present study was to investigate the longitudinal pattern of initial insulin responses during sequential IVGTTs from the onset of islet autoimmunity. We present the results of an analysis comparing FPIR values in children who had multiple islet autoantibodies and developed clinical diabetes during the follow-up and in children who remained healthy but were positive for classical islet-cell antibodies (ICA) alone, a group which actually has been shown to be at a low risk for developing type 1 diabetes (17).

## Subjects and methods

### Study design

The Finnish DIPP Study, launched in 1994, is an ongoing population-based prospective study in Turku, Oulu and Tampere University Hospitals, Finland. In the DIPP Study, cord blood is used for screening infants for HLA-conferred risk for type 1 diabetes (18). Families with a baby carrying HLA risk alleles are invited for follow-up starting when the infant is 3 months old until at least to the age of 15 years. The children are regularly tested for signs of β-cell autoimmunity. Follow-up visits are scheduled every 3–6 months until the age of 2 years and every 6–12 months thereafter. If a child develops islet autoantibodies, the follow-up interval becomes 3 months until the study endpoint is reached.

### Study participants

Characteristics of the study children are presented in Table 1. Originally, a series of children (*n*=685) with at least one IVGTT performed after the initial appearance of islet autoantibodies during the years 1995–2013 was compiled from the three clinical centres. We selected two groups for the current analyses: i) children who had multiple autoantibodies and progressed to type 1 diabetes by the end of June 2013 (progressors; *n*=210) and ii) children who remained healthy during the follow-up and carried a relatively low risk of type 1 diabetes – based on persistent or transient positivity for ICA only from the time of the initial seroconversion without other emerging autoantibodies during follow-up (non-progressors; *n*=192). The 283 children with other autoantibody combinations and remaining non-diabetic were not included in the current analyses.

The autoantibody status was determined based on samples analysed by the beginning of August 2013. Children were diagnosed with type 1 diabetes based on the World Health Organization (WHO) criteria (19). In addition to index children that were observed from birth, 17 siblings were included in the group of progressors with a median follow-up time of 2.92 years (range 0.45–9.30 years) before diagnosis. Part of the study children (*n*=155, 38.6% of all) were involved in the DIPP nasal insulin trial which showed no protective effect by intranasal insulin (20). There were no differences in the baseline FPIR (*P*=0.29) or in the changes from the baseline FPIR to the last FPIR (*P*=0.81) between the placebo and nasal insulin groups in the DIPP intervention trial. Accordingly, their data were included in the current analyses. The seroconversion age was younger and the follow-up time shorter among the progressors compared to non-progressors (Table 1).

The present study was conducted according to the guidelines of the Declaration of Helsinki, and was approved by the Joint Commission on Ethics of Turku University and Turku University Central Hospital. Written informed consent was obtained from all subjects and/or their guardians.

### β-cell autoimmunity

Four types of islet autoantibodies were analysed: ICA, insulin autoantibodies (IAA), antibodies to glutamic acid decarboxylase (GADA) and tyrosine phosphatase-related insulinoma-associated 2 molecule (IA-2A) (17, 21). The autoantibody analyses and cut-off limits have been described earlier (21).

### Intravenous glucose tolerance tests

IVGTTs were performed in children with islet autoantibodies to evaluate β-cell function. The tests in this study were performed between May 1996 and May 2012 according to a standard protocol (22). Briefly, after overnight fasting and the use of local anaesthetic (EMLA, AstraZeneca), the fasting samples were drawn through an i.v. cannula at 5 and 0 min before the start of the glucose infusion. The glucose dose (0.5 g/kg, maximum 35 g) was infused through the cannula in 3 min±15 s, and samples were subsequently taken at 1, 3, 5 and 10 min after the end of the infusion. The recommended interval for IVGTTs in autoantibody positive DIPP children was every 6–12 months.

### Analyses of plasma glucose and serum insulin

Plasma glucose concentrations were measured by an enzymatic method (23). For the quantitative serum insulin measurements an ELISA (Insulin ELISA Kit, Dako, Glostrup, Denmark) method (24) was used in the Oulu centre for samples obtained in Oulu and Tampere. In Turku, the method for serum insulin measurement has changed twice during the DIPP Study. From the beginning of the study until March 2001 a RIA (Phasadeph, Pharmacia) was used. Between March 2001 to November 2004 a time-resolved immunofluorimetric assay (TR-IFMA, Autodelfia, Wallac, Turku, Finland) was applied. The currently used method is an electrochemiluminescence immunoassay (ECLIA, Roche Diagnostics). The changes in the insulin concentrations have been small when comparing the different methods used in Turku: there was an increase by 1% after changing to TR-IFMA and a decrease by 3% since the change to ECLIA.

We have previously compared the insulin assays in the Oulu and Turku laboratories (16) and used a regression transformation equation to make the values from the Oulu laboratory comparable with the Turku RIA values. We also corrected the insulin concentrations obtained with the more recent Turku assays to make them comparable with the concentrations generated with the initial Turku RIA.

### Variables used to assess glucose metabolism

β-cell function was evaluated by FPIR and the area under the 0–10 min insulin curve (AUC_0–10 min_ for insulin). As surrogate markers for insulin sensitivity, fasting insulin concentrations, the insulin resistance index determined by the homeostasis model assessment (HOMA-IR) (25) and the HOMA-IR to FPIR ratio (relative insulin resistance) were used. Fasting values for insulin and glucose were calculated as the mean of −5 and 0 min values from the IVGTT. When the fasting insulin values were below the detection limit, that limit was used as an actual value. Glucose values at 60 min from the Turku data set were analysed to investigate the late-phase glucose concentrations.

### Statistical analysis

The response variables (glucose metabolism markers) were log-transformed for the analyses. Time periods of 0–2, 2–4 and 4–6 years before diagnosis (progressors) or the last IVGTT (non-progressors) were analysed separately. The progressors' last response variable before diagnosis (median 2.86 years before diagnosis; range from 5 days to 14 years) was compared with the previous one by a paired samples *t*-test. The median time between the last two measurements was 1.05 years (range: 0.23–4.71 years). Owing to skewed distribution of the age at seroconversion and follow-up time, the comparisons between the study groups were performed with the Mann–Whitney *U* test.

The scatterplots between age and response variables were noisy, so the data was explored using cubic splines (26) to smooth curves in order to reveal the mean or median response profile. To study the possible early differences between the two groups these analyses were also performed excluding data from last 2 years prior to diagnosis in the progressors. The patterns for females and males appeared similar and the combined profiles are shown.

The effect of age on response variables was assessed by a linear mixed model. Predictor variables were age, group and their interaction. Given estimates for age represent how response variables change when age is increased by 1 year. Study variables between the study groups were compared at the ages of 2, 4, 6, 8 and 10 years. In the age-dependent comparison, the difference between the study groups describes how many percent the response variable has changed in non-progressors compared to progressors.

Statistical analyses were performed with Statistical Analytical Software (SAS, version 9.3, SAS Institute, Cary, NC, USA) and Statistical Package for the Social Sciences (SPSS, version 21, IBM Corp., Armonk, NY, USA). Cubic splines were drawn using SAS GPLOT with SM30 interpolation parameter. *P* values of <0.05 were considered statistically significant.

## Results

### Metabolic changes before the diagnosis of type 1 diabetes

**Figure 1:**
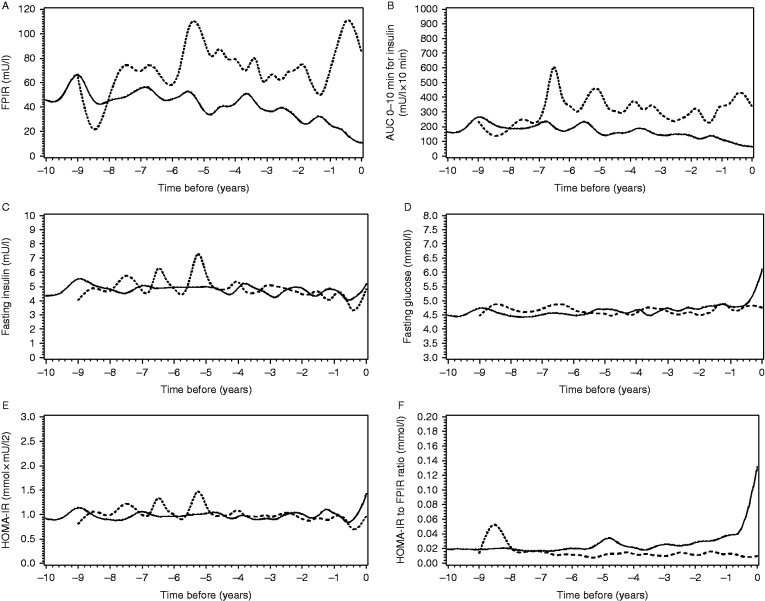
Median values of the study variables ((A) FPIR, (B) AUC_0–10 min_ for insulin, (C) fasting insulin, (D) fasting glucose, (E) HOMA-IR index and (F) HOMA-IR to FPIR ratio) before the diagnosis of type 1 diabetes in the progressors (black line) and in the non-progressors until the last IVGTT (dotted line). Point 0 indicates the time of the diagnosis or the last IVGTT. The *x* axis indicates years before the diagnosis or the last IVGTT. (A and B) The *y* axis indicates the unit for the study variable. FPIR and AUC_0–10 min_ for insulin were decreased 0–2 and 2–4 years (*P*<0.001 for both variables and time periods) and up to the time period of 4–6 years before the diagnosis as compared to the non-progressors (*P*=0.001 and *P*=0.002 respectively). (C) Fasting insulin did not differ between the groups before the diagnosis. (D) Fasting glucose differed between the groups 0–2 years before the diagnosis (*P*=0.008). (E) HOMA-IR index did not differ between the groups before the diagnosis. (F) HOMA-IR to FPIR ratio was increased in the progressors during the time periods 0–2, 2–4 and 4–6 years prior to diagnosis (*P*<0.001, *P*<0.001 and *P*=0.005 respectively).

FPIR and AUC_0–10 min_ for insulin were decreased 0–2, 2–4 and 4–6 years before the diagnosis in the progressors as compared to the non-progressors (*P*<0.005, Fig. 1A and B). There was no difference in the fasting insulin concentrations between the groups (Fig. 1C). Fasting glucose concentrations increased during the last 6 months prior to diagnosis in the progressors (difference between the groups during 0–2 years prior to diagnosis; *P*<0.01), while the groups did not differ from each other during the earlier time periods (Fig. 1D). HOMA-IR index did not differ between the groups before the diagnosis (Fig. 1E). HOMA-IR to FPIR ratio was increased in the progressors 0–2, 2–4 and 4–6 years before the diagnosis (*P*<0.01; Fig. 1F).

In the last two samples before diagnosis, the change in FPIR, AUC_0–10 min_ for insulin, fasting glucose, HOMA-IR and the HOMA-IR to FPIR ratio reached significance (*P*<0.005; Supplementary Fig. S1A–B, D–F) whereas the increase in fasting insulin did not reach significance (*P*=0.07; Supplementary Fig. S1C).

There were no significant differences in the glucose metabolism markers when comparing progressor children who had different primary autoantibodies (IAA, GADA or IA-2A) at seroconversion (Table 2).

### Longitudinal age-dependent comparisons between the study groups

**Figure 2:**
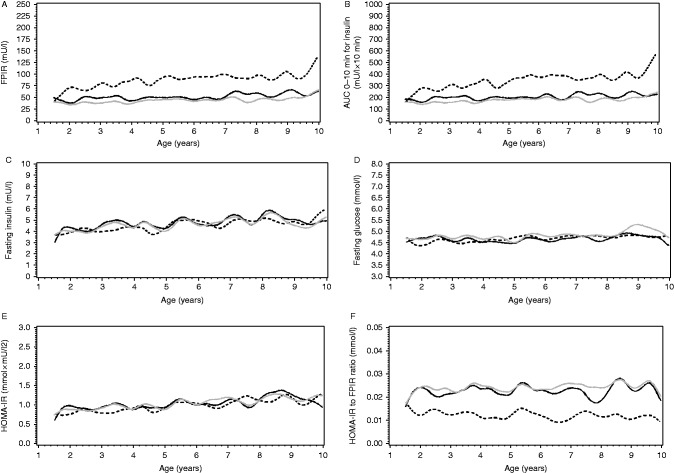
Mean values of the study variables ((A) FPIR, (B) AUC_0–10 min_ for insulin, (C) fasting insulin, (D) fasting glucose, (E) HOMA-IR index and (F) HOMA-IR to FPIR ratio) in cubic splines between the non-progressors and progressors as a function of age (years). The solid line shows the values of the progressors. The black line represents the values when the last 2 years prior to diagnosis were excluded. The grey line represents the values when the last 2 years prior to diagnosis were included. The black dotted line represents the non-progressors. (C, D and E) Fasting insulin increased 3.9%/year, fasting glucose 0.50%/year and HOMA-IR index increased 4.4%/year (*P*<0.001, *P*=0.0024 and *P*<0.001 respectively).

The difference in FPIR between the progressors and non-progressors was significant in all age groups (*P*<0.001). FPIR was higher in the non-progressors than in the progressors, and the difference increased with age (at 2 years: difference 50% (95% CI 28–75%) and at 10 years: difference 172% (95% CI 128–224%); Fig. 2A). AUC_0–10 min_ for insulin showed a similar difference between the groups (*P*<0.001; at 2 years: difference 36% (95% CI 17–58%) and at 10 years: difference 186% (95% CI 143–237%); Fig. 2B).

The effect of age was similar in both study groups for fasting insulin and fasting glucose and HOMA-IR (the interaction between age and group was for these variables *P*=0.11, *P*=0.16 and *P*=0.08 respectively). Fasting insulin increased 3.9% (95% CI 2.5–5.2%) per year, fasting glucose 0.50% (95% CI 0.17–0.83%) per year and HOMA-IR index increased 4.4% (95% CI 2.9–5.9%) per year (*P*<0.005; Fig. 2C, D and E). HOMA-IR to FPIR ratio was lower in the non-progressors than in the progressors at all ages (*P*<0.001; at 2 years: difference 46% (95% CI 36–54%) and at 10 years: difference 60% (95% CI 52–67%); Fig. 2D). There was no systematic effect of age on this variable because both the FPIR and HOMA-IR changed similarly with age.

#### Glucose at 60 min

**Figure 3:**
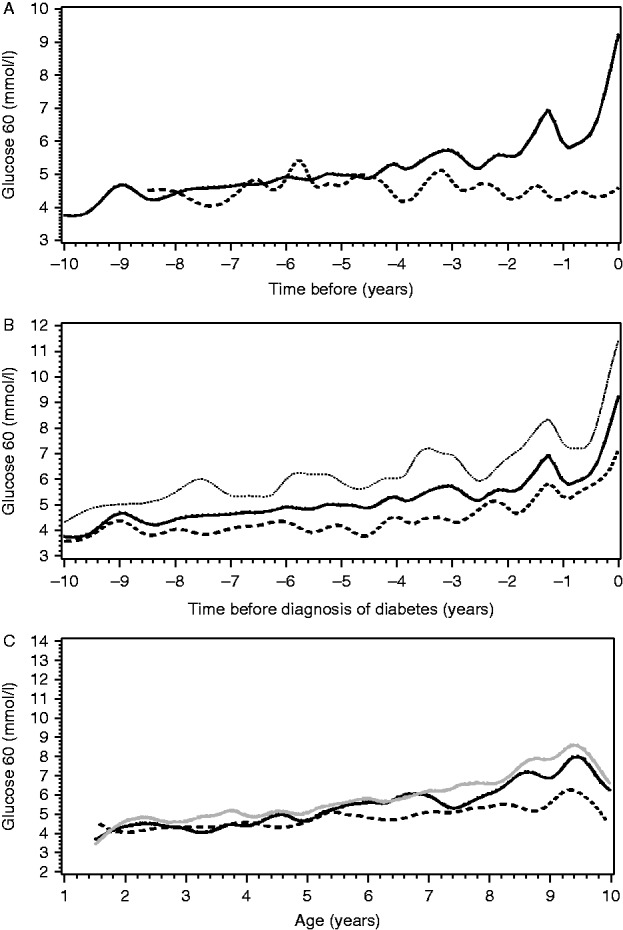
(A) Median 60-min values of glucose before the diagnosis of type 1 diabetes in the progressors (black line) and in the non-progressors until the last IVGTT (dotted line). Point 0 indicates the time of the diagnosis or the last IVGTT. The *x* axis indicates years before the diagnosis or the last IVGTT. The *y* axis indicates plasma glucose concentration at 60 minutes. (B) The median, upper and lower quartile for 60-min glucose values before the diagnosis of type 1 diabetes. Point 0 indicates the time of diagnosis. The *x* axis indicates years before the diagnosis. The *y* axis indicates plasma glucose concentration at 60 minutes. For other variables in this study, the quartiles before the diagnosis of type 1 diabetes are seen in the Supplementary File. (C) Mean values of glucose at 60 min in cubic splines among the non-progressors and progressors as a function of age (years). The solid line shows the values of the progressors. The black line represents the values when the last 2 years prior to diagnosis were excluded. The grey line represents the values when the last 2 years prior to diagnosis were included. The black dotted line represents the non-progressors. Glucose values at 60 min were obtained from the Turku data set (299 samples from non-progressors and 325 samples from progressors).

By using the Turku data set only, the glucose at 60 min was increased in the progressors 0–2 and 2–4 years before the diagnosis (*P*<0.001 and *P*=0.03 respectively; Fig. 3A). The change in glucose at 60 min was significant in the last two samples before diagnosis (*P*<0.001, Fig. 3B). Glucose at 60 min was lower in non-progressors from the age of 6 years onwards and the difference increased with age (*P*<0.001; at 6 years: difference 11% (95% CI 7–15%) and at 10 years: difference 24% (95% CI 18–29%); Fig. 3C).

## Discussion

The results of this study show that β-cell function is reduced years before the diagnosis in children who progress to type 1 diabetes. The difference in FPIR between the progressors and non-progressors was evident 4–6 years before the diagnosis. In age-dependent longitudinal comparison, FPIR was constantly lower in the progressors than in the non-progressors, even when the FPIR values from the last 2 years prior to diagnosis were excluded from the analysis. The difference between the study groups increased with age: the mean FPIR was 2.7 times greater in the non-progressors than in the progressors at the age of 10 years. These findings imply that children at risk fail to increase their β-cell function adequately to maintain glucose homeostasis with age and increasing body size. Interestingly, in an earlier study ICA-negative siblings of patients with type 1 diabetes were described to have significantly lower FPIR from 8 years onwards when compared to control children (27).

The control group in this study (non-progressors) comprised children who tested positive for the classical ICA only. We have shown earlier that single temporary or persistent ICA positivity is associated with a 5-year cumulative risk of type 1 diabetes of only 1.4 and 4.7%, respectively (17), which is rather similar to risk of Finnish children with no islet autoantibodies. The median seroconversion age of the non-progressors was 4.6 years, whereas the progressors seroconverted earlier, at the median age of 1.6 years, and developed multiple autoantibodies later. It is known that the presence of two or more autoantibodies substantially increases the risk of progression to clinical type 1 diabetes with a 5-year cumulative risk being around 40%; in a 15-year follow-up the risk is over 80% (28).

The baseline FPIR of the progressors and non-progressors appears similar (Fig. 2A). However, no data from the first year of life was available for comparison. As the progressors seroconverted early, the low level of FPIR suggests a rapid autoimmune-mediated decline in β-cell function shortly after the appearance of autoantibodies. However, it is also possible that FPIR in the progressors was already low prior to this. During early childhood, β-cell mass grows rapidly by replication (29, 30). A high risk of type 1 diabetes could be associated with an already decreased β-cell mass at birth (31). As FPIR represents the response to glucose, it is also possible that β-cells have impaired glucose sensitivity in children at risk to develop type 1 diabetes (32, 33).

In both study groups, fasting insulin, fasting glucose and HOMA-IR index increased by age. Insulin resistance measured by HOMA-IR index does not appear to play a major role in the development of type 1 diabetes, which is in line with our earlier study in which the focus was on the predictive role of FPIR after the appearance of multiple islet autoantibodies (13). The difference between the groups in relative insulin resistance as assessed by HOMA-IR to FPIR ratio in this study was caused by the low FPIR in the progressors.

We also performed a separate analysis in the Turku cohort, where the glucose at 60 min was increased in the progressors 2–4 years before the diagnosis. In the retrospective comparison in our earlier study (34) the OGTT-derived 2-h plasma glucose and random plasma glucose values were significantly different from those 1.5 years before diagnosis; thus, it seems these increased late phase glucose values in IVGTT precede abnormalities of OGTT before the diagnosis.

The strengths of this study include the long follow-up and the repeated monitoring of autoantibodies and IVGTTs in a large cohort of children observed from birth. The variation in the number and timing in the IVGTTs is a limitation of the current work but that was unavoidable in the young children.

To conclude, our study demonstrates that the insulin secretory capacity of the β-cells is compromised early during the disease process leading to type 1 diabetes. The low insulin response to glucose seems to be an essential feature of the disease process and suggests that children progressing to type 1 diabetes have an intrinsic impairment in their β-cell mass or function that further deteriorates under environmental pressure.

## Author contribution statement

M K Koskinen, O Simell, J Toppari and R Veijola contributed to the study design. M K Koskinen, O Helminen, S Aspholm, M Mäkinen, T Simell, J Ilonen, M Knip, R Veijola and O Simell contributed to the data acquisition. J Matomäki and M K Koskinen analysed the data. M K Koskinen, O Helminen, J Matomäki, J Mykkänen, M Vähä-Mäkilä, R Veijola, J Toppari and O Simell contributed to the interpretation of data. M K Koskinen, O Helminen, S Aspholm, J Mykkänen, M Mäkinen, V Simell, T Simell, J Ilonen, M Knip, R Veijola, J Toppari and O Simell contributed to the drafting of the work. M K Koskinen, O Helminen, J Matomäki, J Mykkänen, M Mäkinen, M Vähä-Mäkilä, V Simell, T Simell, M Knip, R Veijola, J Toppari and O Simell critically revised the manuscript for important intellectual content. M K Koskinen and J Matomäki are the guarantors of this work and, as such, had full access to all of the data in the study and take responsibility for the integrity of the data and the accuracy of the data analysis.

## Figures and Tables

**Table 1:** Characteristics of the study children.

**Clinical characteristics**	**Non-progressors**^a^	**Progressors**^b^	***P* value**
Number of subjects	192	210	
Sex, male (%)	116 (60.4%)	117 (55.7%)	0.34^c^
Age at seroconversion (any autoantibody, age in years)^d^, median (min–max)	4.59 (0.65–12.60)	1.55 (0.51–10.59)^e^	**<0.001**^f^
Age when persistently positive for two autoantibodies (years)^d^, median (min–max)	–	2.02 (0.80–10.59)^e^	
Age at diagnosis (years), median (min–max)	–	6.58 (1.63–16.11)	
Follow-up time (years), median (min–max)	12.02 (2.45–15.54)	5.49 (0.45–16.09)	**<0.001**^f^
Number of IVGT tests	318g,h	661h,i	

a Non-progressors tested positive for islet-cell antibodies (ICA) only and did not develop clinical disease during the follow-up.

b Progressors are children with multiple (≥2) islet autoantibodies who subsequently progressed to type 1 diabetes.

c *χ*^2^ test.

d Seroconversion to autoantibody positivity was defined based on positivity for a minimum of one autoantibody in at least two consecutive samples. Persistent positivity for multiple (≥2) autoantibodies refers to positivity for a minimum of two autoantibodies in at least two consecutive samples.

e *n*=193, there were 17 siblings among the progressors whose follow-up started later than at birth. As their seroconversion date was not clearly defined, they were not included in these specific analyses.

f Mann–Whitney *U* test.

g Tests were performed in the Turku centre.

h Analyses of glucose at 60 min were performed with the data from the Turku centre (299 samples from non-progressors and 325 samples from progressors).

i A total of 326 IVGTTs were performed in the Oulu and Tampere centres and 335 IVGTTs in the Turku centre.

**Table 2:** Mean and median values of the FPIR, AUC_0–10 min_ for insulin, fasting glucose and AUC_0–10 min_ for glucose at the time of 2 (±1) years prior to diagnosis in progressor children when single autoantibody (IAA, GADA or IA-2A) at seroconversion could be determined.^a^^,^^b^

**Glucose metabolism markers**	**GADA**	**IA-2A**	**IAA**	***P***^c^
*n*	27	8	36	
FPIR				
Mean (s.d.)	48 (51)	48 (27)	34 (19)	0.60
Median (min–max)	36 (11–274)	46 (9–92)	31 (11–86)	
AUC_0–10 min_ for insulin				
Mean (s.d.)	176 (197)	201 (133)	143 (68)	0.99
Median (min–max)	123 (51–1031)	171 (33–465)	128 (54–316)	
Fasting glucose (mmol/l)				
Mean (s.d.)	4.8 (0.4)	4.9 (0.6)	4.7 (0.6)	0.22
Median (min–max)	4.8 (3.8–5.5)	5.0 (3.8–5.8)	4.7 (3.3–6.0)	
AUC_0–10 min_ for glucose				0.40
Mean (s.d.)	168 (17)	169 (24)	161 (14)	
Median (min–max)	170 (138–196)	169 (156–210)	162 (136–194)	

a ICA was not included in this spesific analysis.

b Children with multipositivity at seroconversion were not included in this analysis as the first autoantibody could not be determined.

c Overall *P* value in one-way ANOVA.
